# Effects of Hypoxic Exposure during Feeding on SDA and Postprandial Cardiovascular Physiology in the Atlantic Cod, *Gadus morhua*

**DOI:** 10.1371/journal.pone.0046227

**Published:** 2012-09-25

**Authors:** Jane W. Behrens, Michael Axelsson, Stefan Neuenfeldt, Henrik Seth

**Affiliations:** 1 National Institute of Aquatic Resources, Technical University of Denmark, Charlottenlund, Denmark; 2 Department of Biological and Environmental Sciences, University of Gothenburg, Gothenburg, Sweden; 3 Department of Neuroscience and Physiology, University of Gothenburg, Gothenburg, Sweden; Institute of Marine Research, Norway

## Abstract

Some Atlantic cod in the Bornholm Basin undertake vertical foraging migrations into severely hypoxic bottom water. Hypoxic conditions can reduce the postprandial increase in gastrointestinal blood flow (GBF). This could subsequently postpone or reduce the postprandial increase in oxygen consumption (MO_2_), i.e. the SDA, leading to a disturbed digestion. Additionally, a restricted oxygen uptake could result in an oxygen debt that needs to be compensated for upon return to normoxic waters and this may also affect the ability to process the food. Long-term cardio-respiratory measurements were made on fed *G. morhua* in order to understand how the cardio-respiratory system of feeding fish respond to a period of hypoxia and a subsequent return to normoxia. These were exposed to 35% water oxygen saturation for 90 minutes, equivalent to the time and oxygen level cod voluntarily endure when searching for food in the Bornholm Basin. We found that i) gastric and intestinal blood flows, cardiac output and MO_2_ increased after feeding, ii) gastric and intestinal blood flows were spared in hypoxia, and iii) there were no indications of an oxygen debt at the end of the hypoxic period. The magnitude and time course of the measured variables are similar to values obtained from fish not exposed to the hypoxic period. In conclusion, when cod in the field search for and ingest prey under moderate hypoxic conditions they appear to stay within safe limits of oxygen availability as we saw no indications of an oxygen debt, or negative influence on digestive capacity, when simulating field observations.

## Introduction

The Bornholm Basin of the central Baltic is characterized by a permanent halocline at approximately 50–60 m depth separating low saline surface water from high saline deep water. Furthermore, major Baltic inflows of heavy, oxygen rich bottom water are rare leaving the oxygen content below the halocline vertically stratified on the scale of meters and the bottom waters almost permanently hypoxic, and, at the greatest depths, even anoxic [1]. Use of archival tags on Atlantic cod (*Gadus morhua*) in the Bornholm Basin has revealed that some individuals undertake vertical migrations into the severely hypoxic bottom water, with the visit time being strongly correlated to the actual degree of hypoxia, for example 90 min at 35% oxygen saturation after which the fish returns to well-oxygenated water [2]. For some individuals the duration of sojourns into water with oxygen saturation ≤20% (oxygen conditions often considered lethal for this species, [3], [4]) even lasted up to an hour. These excursions are most likely related to foraging as indicated by the presence of fresh benthic food items in the stomachs of Baltic cod caught in pelagic hauls [5]. Not only Baltic cod but also Atlantic tuna show movements into hypoxic waters probably as a direct consequence of a limited amount of food in normoxic waters [6], and in such situations the individual has to search for food in what is to be considered as adverse environmental conditions.

Feeding induces several cardio-respiratory changes that enable the animal to efficiently digest, absorb, and redistribute the nutrients. Gastrointestinal blood flow (GBF) increases profoundly to facilitate digestion and absorption of the food. However, under circumstances of low oxygen availability regional blood flow must be altered, and in unfed fish there is a redistribution of blood away from the gastrointestinal area [7], [8], [9]. Thus, if GBF is curtailed while the fish search for and ingest prey in hypoxic water this may impact digestive performance, which may continue following the return to normoxic waters. This could either be manifest through prolonged gastric evacuation time or by reduced assimilation efficiency. It may on the contrary be that there is no redistribution of gastrointestinal blood during hypoxia, as shown in fed sea bass where the relative proportion of cardiac output reaching the gut did not decrease under hypoxic conditions (although absolute gut blood flow did) [10]. Furthermore, if the fish face the limitations of a reduced metabolic scope (the metabolic scope being the difference between the maximum aerobic metabolic rate and the standard metabolic rate [SMR], representing the metabolic confines within which all aerobic activities must be undertaken [11] it may have to rely partly on anaerobic energy production which will result in excess post hypoxic oxygen consumption (EPHOC) [12, 13]. This could interfere with the cardiovascular provision to the gut. Considering that the ambient oxygen saturation below which basal requirements can no longer be met (S_crit_) is higher in post-prandial compared to unfed fish [14] it may well be that the fish must resort to glycolytic metabolism well before encountering waters with 16 to 23% oxygen saturation, that is, the S_crit_ for non-digesting cod at 5–10°C [15].

Consequently, to assess the energetic significance of feeding in hypoxic waters we need to understand how the cardio-respiratory system of feeding fish responds to a period in hypoxia followed by return to normoxia. The aim of the present study was therefore to investigate whether ingestion of prey and initiation of digestion in moderately hypoxic water will i) leave GBF maintained or curtailed, where the latter may impact digestive performance subsequent to a return to normoxic waters, and/or ii) whether the same circumstances will results in an oxygen debt which has to be paid back following return to normoxic water. This was done by long-term cardio-respiratory measurements before and following feeding in *G. morhua* exposed to 90 min of 35% of oxygen saturation (corresponding to the time and oxygen level cod voluntarily endure when searching for food in the Bornholm Basin [2] and thereafter returned to normoxia. The experimental temperature was 6.5°C, corresponding to the average of summer temperatures encountered by *G. morhua* in that area [5].

## Materials and Methods

### Experimental Animals

Juvenile Atlantic cod (n = 35, weight range 470–920 g) were caught by hook in Skagerrak (10°E;58°N) and transported to the University of Gothenburg in a 200 L thermo-isolated tank with 10°C water that was continuously oxygenated. Upon arrival the fish were kept in 4 m^3^ fiberglass tanks supplied with aerated seawater (10°C) from a recirculating water system and a photoperiod of 12∶12 h light-dark conditions. The fish were acclimatized for a minimum of four weeks before experiments were initiated. The fish were fed two times a week with chopped pollock.

### Ethics Statement

Ethical permit 13/2007 from the Animal Ethics Committee of Gothenburg covered all experiments reported here.

### In Vivo Surgical Procedures

Fish were fasted for approximately 1 week prior to surgery. Individual fish were anesthetized in water containing 0.133 g l^−1^ 3-aminobenzoicacid ethyl ester (MS-222) buffered with sodium bicarbonate (0.30 g l^−1^) and transferred to an operating table covered with soft water-soaked rubber foam. The gills were continuously irrigated with aerated saltwater (10°C) containing MS-222 (0.066 g l^−1^) buffered with sodium bicarbonate (1.5 g l^−1^).

Relative cardiac output (CO) was measured with a custom-made flow probe on a Doppler transducer (Iowa Doppler Products, Iowa, USA), with a diameter between 2.5 and 3.5 mm and placed on the ventral aorta. To place the cuff, the fish was positioned on its left side, and a small incision was made at the base of the fourth gill arch dorsal to the ventral aorta. Connective tissue was removed until the ventral aorta was visible and there was enough room for the cuff. The cuff lead was secured with one suture close to the cuff and one to the back of the fish. To measure relative gastric and intestinal blood flow, Doppler flow probes with diameters between 1.1 and 1.2 mm were placed around, respectively, the coeliac (CA) and the mesenteric artery (MA) roughly 5 mm past the bifurcation of the coeliacomesenteric artery. To access the two arteries, the fish was placed on its left dorsolateral side, and a 25-mm incision was made ventrodorsally from the base of the pectoral fin. The vessels were dissected free using blunt dissection, taking care not to damage any of the surrounding nerves. Once the cuffs were positioned, the leads were secured with sutures to the skin, and the incision was closed with uninterrupted sutures.

### Metabolic Oxygen Consumption (MO_2_)

The fish was placed in a closed respirometer (7.9 L) custom-made from perspex submersed in an outer 240 L tank supplied with a continuous flow of aerated seawater and covered to minimize visual disturbance of the fish. Circulation of water through the respirometer was achieved by two submersible pumps, one working continuously to ensure mixing inside the respirometer and one controlled by a time relay to close the respirometer for 10 min during MO_2_ measurements. The relative change in oxygen tension/partial pressure of oxygen in the water was measured using an oxygen-meter (Oxi 340 i, WTW, Weilheim, Germany) placed in-line with the mixing pump.

After each experiment, the chamber was thoroughly cleaned using hot water to remove any microbial growth. Background oxygen consumption was measured in an empty respirometer.

### In vivo Recordings

After surgery animals were allowed to recover within the respirometer at 6.5°C for at least 24 h prior to the experiment. After this period the fish was connected to the Doppler flow meter and once a stable baseline had been attained, routine baseline MO_2_ and cardiovascular variables were recorded for 3–4 h. Each fish was then lightly anaesthetized in buffered MS-222 (100 mg l^−1^, Sigma-Aldrich) until righting reflexes were lost, then fed by gavage a ration of 2% of their body mass of pollock. One group (n = 7, body mass 650±61 g) was allowed to digest the food under normoxic conditions whereas another group (n = 7, body mass 660±55 g) was exposed to 35% oxygen saturation (relative to air) for 90 minutes 2 h post-feeding and then returned to normoxia for the remainder of the measuring period. Two further groups (both n = 5, body masses 688±52 and 674±47 g, respectively) were sham fed and treated as the two groups described above, except that the measured parameters were only followed for 10 h post sham-feeding, however at a higher resolution with one measuring point every 10 min as compared to every 20 min in the force-fed groups. The variable effect of sham-feeding on the measured cardio-respiratory variables had subsided after 2 h. Therefore, the first 2 h of postprandial data are not included in the present analysis.

During the period with low oxygen saturation, water in the respirometer was recycled through a gas-exchange column, and oxygen concentration was continuously recorded using an oxygen regulator system (Lolioxy, Loligo Systems) that controlled a flow of 100% nitrogen into the column via a solenoid valve. The desired oxygen saturation level (35%) was attained within a 15-min period. Cardiovascular parameters and rate of oxygen consumption (MO_2_) (determined for a 10-min interval every 20 min) were recorded for up to 75 hr post feeding. At the end of the experiment, fish were euthanized by an overdose MS-222 followed by a sharp blow to the head.

### Data Acquisition

The Doppler flow probes were connected to a directional pulsed Doppler flow meter (model 545C-4; The University of Iowa, Iowa City, USA). MO_2_ (mg O_2_ kg^−1^ h^−1^) was determined from the slope of a linear regression of the decline in PO_2_ over the 10 min measuring period, using the formula:
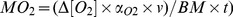
(1)where Δ[*O_2_*] is the relative difference in oxygen partial pressure (%) before and at the end of the 10 min period when the respirometer was closed as calculated by the slope of the resultant linear regression; α_O2_ is the saturated oxygen content (mg l^−1^) of water at the particular temperature and barometric pressure; *v* is the volume of the closed circulation excluding the volume of the fish, *BM* is the body mass of the fish and *t* is the time. In both cases a PowerLab system connected to a PC running Chart 6 (AD Instruments, Castle Hill, Australia) was used for the analog/digital conversion.

### Data Analysis and Statistics

Heart rate (HR) was obtained from the phasic flow traces. Due to the relative flow values obtained with a Doppler flow probe, in vivo blood flows (CO, Q_ce_ and Q_me_) are presented as relative changes with the baseline set to 100%. Excess post-hypoxic oxygen consumption (EPHOC) following 90 min of hypoxia at 35% O_2_ saturation was calculated according to Svendsen et al. 2011. The specific dynamic action (SDA, the energy expended on all activities of the body incidental to the ingestion, digestion, absorption, and assimilation of a meal [16]; response was analyzed by comparing the baseline MO_2_ from each fish during a pre-absorptive period (3–4 h before feeding) to the consecutive postprandial MO_2_ data averaged over 1-h intervals. Four variables of the SDA response were quantified: (1) the actual SDA, that is the total energy used; 2) the SDA coefficient, SDA_coef_ (the energy expended relative to the energy ingested); 3) the peak metabolic rate (MO_2peak_), i.e. the maximum oxygen consumption observed averaged over 1 h during the SDA course; 4) SDA duration, which is the duration from the initial increase in postprandial MO_2_ until it is no longer significantly higher than pre-feeding values. Oxygen consumption was converted to energy using an oxycalorific coefficient of 14.06 kJ g O_2_^−1^ [17]. Two sample location tests of the null hypothesis that the means of two responses measured are equal were conducted using Welch's t test which is an adaptation of Student's t-test intended for use with two samples having possibly unequal variances. In the following, the resulting test statistic is labeled as ‘t*’*. The normality assumption for Welch’s t test and the one-way ANOVA (test statistic labeled ‘F’), conducted to test the group effect on mean body mass, was tested using the Shapiro-Wilk test. Testing was done using the GNU software package R (www.r-project.org). Significance was accepted at P<0.05. All values are mean ± SE unless otherwise stated.

For the normoxic fish, the relationships between CO and Q_ce_ and Q_me_, respectively, were assessed by comparing the difference between postprandial CO from pre-feeding levels (ΔCO) with the difference in postprandial Q_ce_ and Q_me_, from baseline (ΔQ_ce_ and ΔQ_me_). The change in minimum postprandial CO from pre-feeding CO levels was plotted against the change in postprandial Q_ce_ from baseline for each block interval, and likewise for Q_me_.

## Results

One-way ANOVA showed that there was no significant difference in mean body mass between the normoxic, the hypoxic or any of the sham fed groups (t = 0.014, df = 3P = 0.898).

### Oxygen Uptake

**Figure 1:**
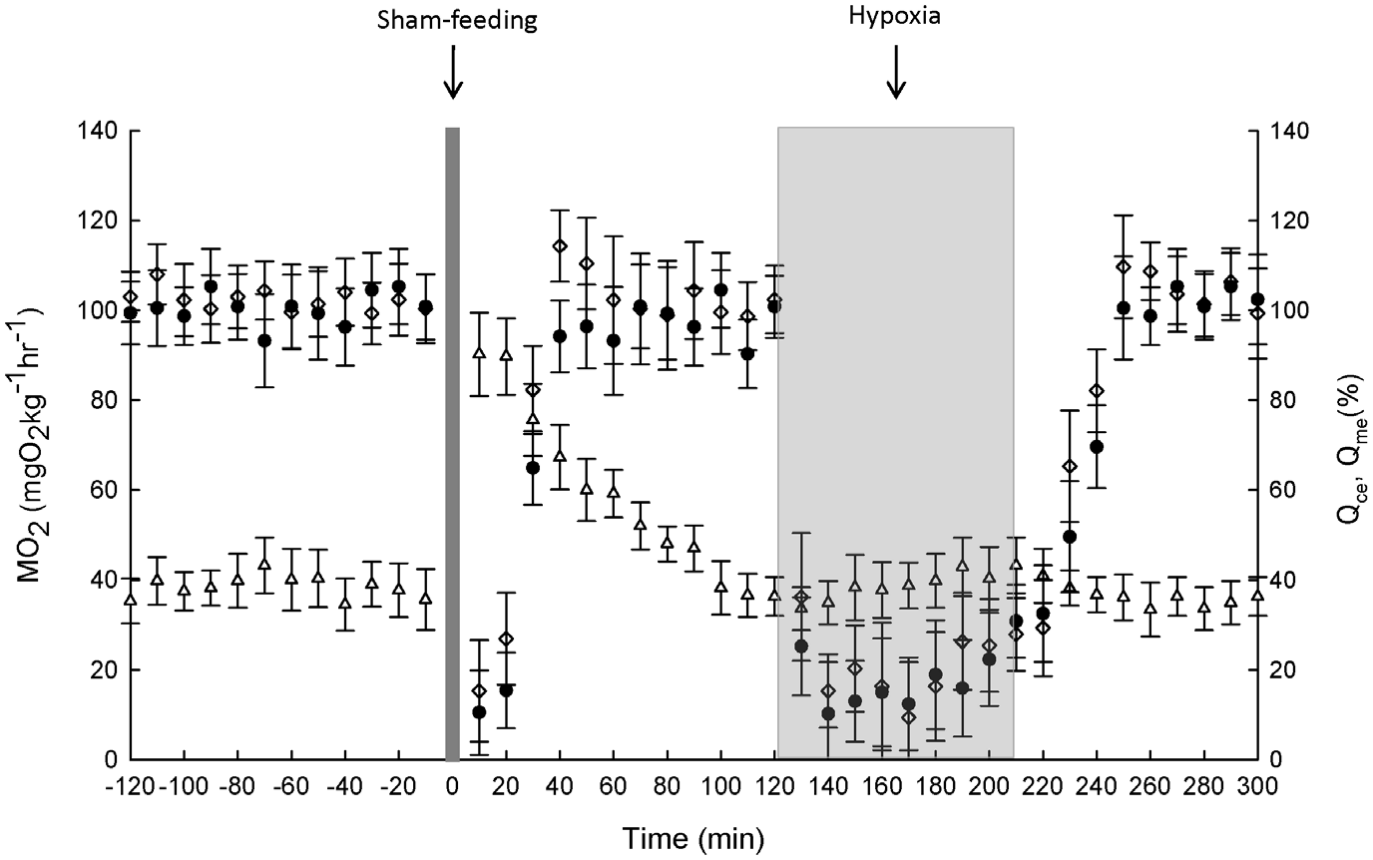
Respiratory and cardiovascular responses to sham-feeding and hypoxic exposure. *G. morhua* were sham-fed at time zero and exposed to 90 min of 35% O_2_ saturation (grey area) 2 hr post sham-feeding. The variables are oxygen uptake (MO_2_, open triangles) and coeliac (Q_ce_, open diamonds) and mesenteric (Q_me_, filled circles) blood flow. Values are mean ± SE, n = 5.

**Figure 2:**
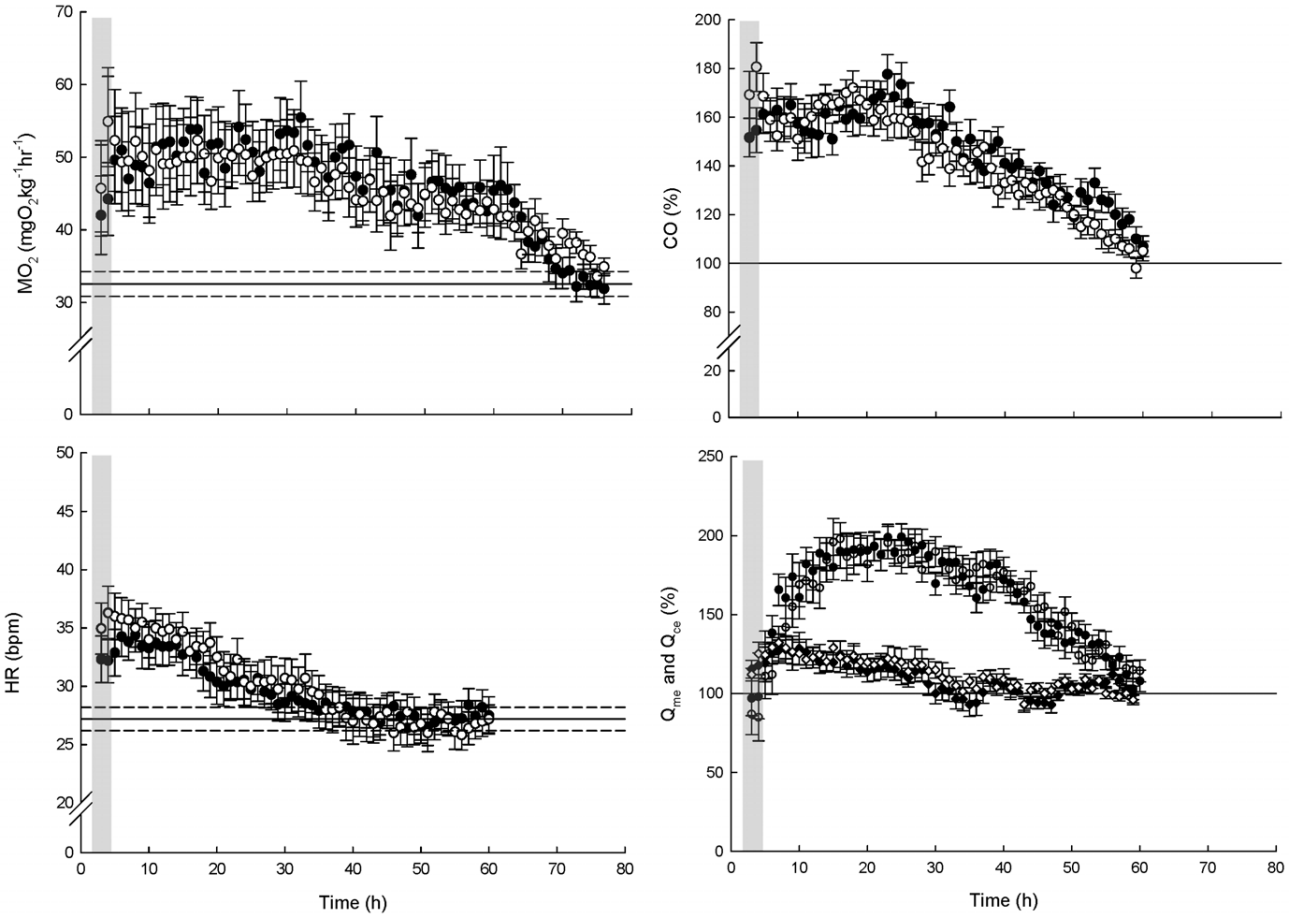
Respiratory and cardiovascular responses to feeding in normoxia and moderate hypoxia. *G. morhua* were force-fed 2% of their body mass at time zero. The variables are oxygen uptake (MO_2_), cardiac output (CO), heart rate (HR) and coeliac (Q_ce_, diamonds) and mesenteric (Q_me_, circles) blood flow. Filled symbols are fish exposed solely to normoxic conditions (n = 7) and open symbols are fish exposed to normoxic conditions except for 90 min of exposure to 35% O_2_ saturation (grey area) (n = 7). All values are mean ± SE. Solid vertical lines indicates pre-feeding baseline values, in case of MO_2_ and HR the dotted lines are ± SE.

The prefeeding MO_2_ in the normoxic and hypoxic group of cod (n = 7 in both) was not significantly different (Table 1). Therefore, these data points were pooled to estimate baseline MO_2_. The MO_2_ of sham-fed fish in normoxia (n = 5) increased abruptly just after sham-feeding, presumably as a result of handling stress. Oxygen consumption then decreased rapidly and returned to baseline values after 90 min and remained at this level for the duration of the measurement. A similar response was seen in fish sham-fed in normoxia (n = 5) and subsequently exposed to a 90 min period of 35% O_2_, although with a slightly increased MO_2_ at the end of the hypoxic period (Fig. 1). Since there was no decrease in MO_2_ during hypoxic exposure, and likewise no EPHOC, it appears that sham-fed fish exposed to 90 min at 35% O_2_ saturation are able to cover their energy needs by aerobic processes. Force-fed fish exhibited the expected SDA response with postprandial MO_2_ being significantly elevated over baseline by 4–5 h postprandial (pooled data pre- and 5 h post-feeding: t = −4.18, df = 13.95, P = 0.0009, Fig. 2). During the course of the SDA, MO_2_ reached a peak value of 54.2±5.9 mg O_2_ kg^−1^ h^−1^ 32 h post post-feeding in the normoxic group and 52.3±5.9 mg O_2_ kg^1^ h^−1^ 17 h post-feeding in hypoxic group (values not significantly different, t = 0.5409, df = 11.348, P = 0.5991), ignoring the slightly higher stress-related MO_2_ obtained during the period of hypoxic exposure (see above). MO_2_ returned to pre-feeding levels after 65 (t = −1.6138, df = 8.1123, P = 0.2186) and 64 h (t = −2.3879, df = 6.421, P = 0.3741) in the normoxic and the hypoxic group, respectively, except one brief subsequent increase in the hypoxic group at 66 h (Fig. 2). The total energy expended on SDA (integration of postprandial MO_2_- prefeeding MO_2_) was very similar between the normoxic and hypoxic groups. Expressing the energy expended relative to the energy ingested (i.e. the SDA_coef_) likewise showed no difference between the two groups. The measured SDA variables are summarized in Table 1. The behavioral response to the hypoxic exposure was somewhat variable, with some individuals becoming completely quiescent while others seemed uneasy at the end of the hypoxic period and initiated movements, although never vigorously.

**Table 1:** Specific dynamic action (SDA) of *G. morhua* at 6–7°C during digestion of 2% body mass pollock diet.

Variable	Normoxic	Hypoxic	t-test
Weight (g)	649.6±61	660±55	t = −0.1311, df = 11.881, p = 0.8979
Pre-feeding MO_2_ (mg O_2_ kg^−1^ h^−1^)	37.5±3.0	33.7±1.7	t = 1.6891, df = 153.341, p = 0.0932
Maximum MO_2_ (mg O_2_ kg^−1^ h^−1^)	54.2±5.9	52.3±5.9	t = 0.5409, df = 11. 348, p = 0.5991
SDA duration (h)	65±12	64±10	t = 0.2971, df = 11.152, p = 0.7853
SDA energy (kJ)	7.4±1.7	7.3±1.1	
SDA coefficient (%)	14.9±3.3	16.1±2.1	

Fish were either exposed solely to normoxic conditions during the SDA (Normoxic) or exposed to 90 min of 35% O_2_ saturation 2 h post-feeding and subsequently returned to normoxic conditions for the remainder of the SDA (Hypoxic). Values are mean±SE and n = 7 in both groups. A p-value greater than 0.05 indicates that there is no difference between the means of the variables.

### Blood Flow Measures

In both groups CO increased rapidly within the first hours after feeding. Notably, CO was further elevated in the fish exposed to 35% O_2_ saturation for 90 min (Fig. 2) being 181±12% at the fourth hour postprandial which however was not significantly higher than in the normoxic group (being 155±11%) (t = 1.9824, df = 11.404, P = 0.07205). At least part of the augmented CO in the hypoxic group was due to an increased HR (Fig. 2). Upon return to normoxia, CO for the hypoxic group decreased to levels comparable to the group that had only experienced normoxia. For the subsequent 20 h CO in both groups oscillated between 150–170% relative to baseline and then reached baseline levels at 54 to 57 h postprandial (Fig. 2). Thus, the time course of postprandial CO was similar between the two groups. While the initial increase in CO (0–25 h) was primarily a result of increased HR (Fig. 2) the increased CO during the remainder of the digestive period (25–60 h) must be, since CO is the product of HR and stroke volume (SV), mediated by an increase in SV.

**Figure 3:**
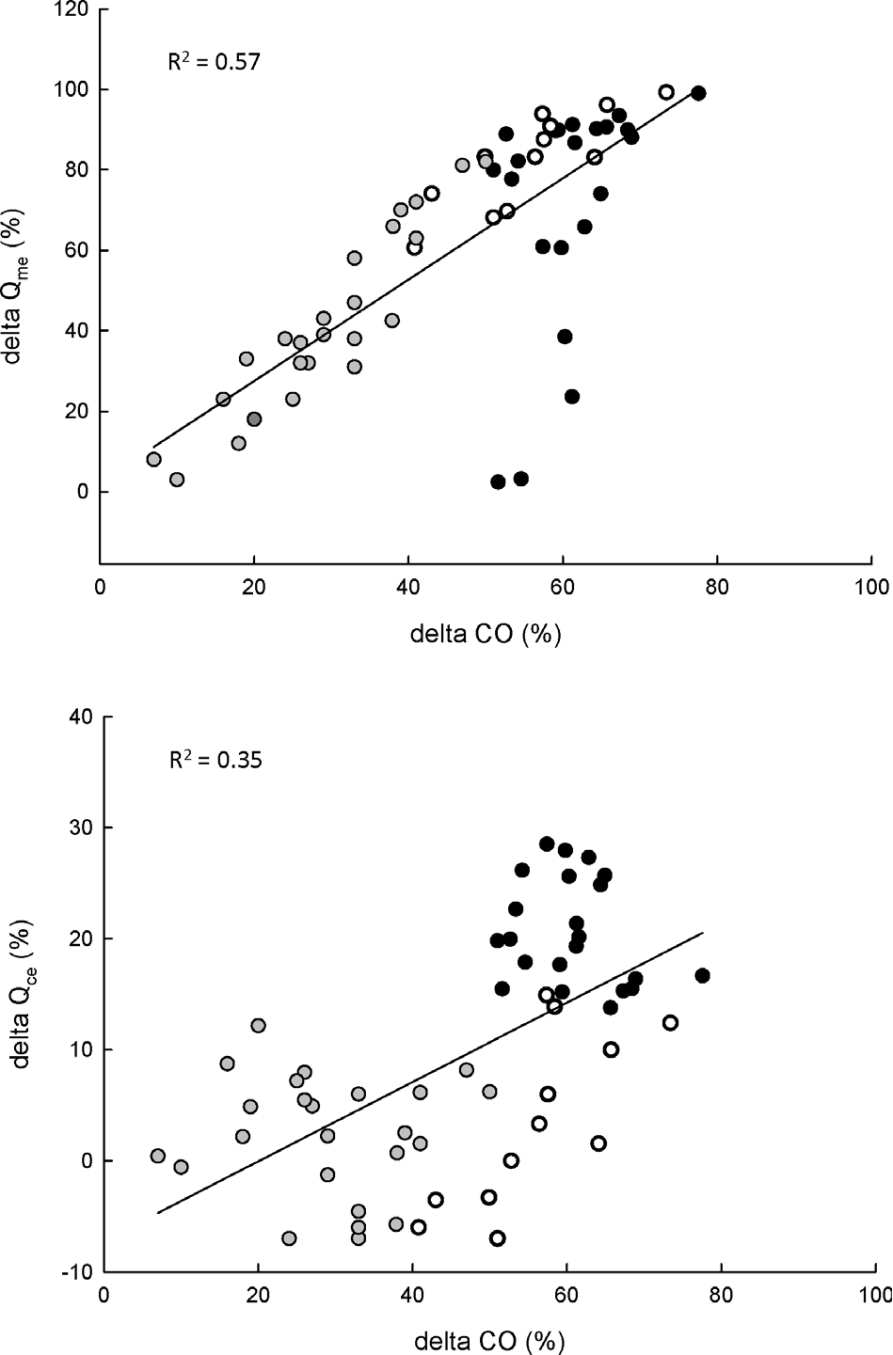
Relationship between postprandial CO and Q_ce_ and Q_me_, respectively. *G. morhua* were force-fed 2% of their body mass and the variables are postprandial changes in respectively mesenteric blood flow (delta Qme) and coeliac (delta Q_ce_) in relation to postprandial changes in cardiac output (delta CO). Data are from fish exposed solely to normoxia, and the black, the white and the grey circles represent values from 3–24 h, 25–36 h and 37–60 h postpranfial, respectively.

In the sham-fed fish gastric (i.e. coeliac, Q_ce_) and intestinal blood flow (i.e. mesenteric blood flow, Q_me_) decreased abruptly both as a result of sham-feeding and hypoxic exposure, but returned to baseline values within 30 to 40 min after sham-feeding and approximately 10 min after exposure to hypoxia, respectively (Fig. 1). In the fed animals there was a clear temporal pattern in the blood flow distribution with an initial increase in the flow through the coeliac artery and a subsequent increase in blood flow through the mesenteric artery (Fig. 2). Q_ce_ had increased significantly (t = −5.2347, df = 6.411, p-value = 0.0015) 6 h after feeding (normoxic group) and 4 hours after feeding for the hypoxic group (t = −4.3969, df = 6.294, p-value = 0.004),reaching peak values of 128% (normoxic group, after 6 hours) and 134% (hypoxic group, after 7 h) (Fig. 2). Q_ce_ returned to baseline after 25 h (normoxic group, t = −1.9152, dt = 6.316, P = 0.1015) and 26 h (hypoxic group, t = - 1.0046, df 0 5, P = 0.3612), respectively. In contrast to the sham-fed group, hypoxia did not decrease Q_ce_ when compared to normoxia, indicating that the blood flow to the stomach is spared when it contains food. Likewise, overall Q_me_ was spared in fed fish exposed to hypoxia (Fig. 2), although with minor reduction in some individuals. Overall postprandial increase in Q_me_ occurred later in both groups compared to Q_ce_. Nevertheless, the response of the Q_me_ far exceeded that of Q_ce_, both in magnitude (near doubling in both groups) and duration. The return of Q_me_ to baseline levels occurred by 56 h postprandial both in the normoxic group (t = −1.5256, df = 7.547, p = 0.1679), and in the hypoxic group (t = −2.4076, df = 5.23, p = 0.05884). Postprandial MO_2_ (i.e. the SDA) remained elevated for 8–9 h longer compared to Q_me_ (Fig. 2). There was a clear linear relationship between the postprandial changes in CO and Q_me_ (Fig. 3) however with no obvious patterns observed in the relationship between changes in CO and Q_ce_ (Fig. 3).

## Discussion

Our results suggest that when cod search for and ingest prey under moderate hypoxic conditions they stay within safe limits of oxygen availability and thus avoid having to resort to glycolytic metabolism, as we saw no indications of an oxygen debt when simulating field observations. Also, with the present level and duration of hypoxia digestive performance can be maintained. In conclusion, these fish appear to forage in nature by exploiting hypoxic waters that do not compromise the response to digestion. Acknowledging however that the capacity of the cardiorespiratory system to accommodate a hypoxic load is not limitless, a greater degree of hypoxia and/or prolonged exposure time (in addition to a larger meal size) may have resulted in negative impact; continuous exposure of *G. morhua* to more severe hypoxia (6–7 kPa at 10°C) has for example been shown to depress postprandial peak MO_2_ with subsequent augmented SDA duration [18].As expected no reflex bradycardia was observed in the present study, the onset of which has been suggested to occur at 20–25% O_2_ sat. for cod held at 8–12°C [19]. This decrease in HR (mediated by a vagal inhibition on the heart) supposedly has several direct benefits to the fish heart working at low oxygen levels and has in cod been shown to contribute to maintenance of overall functional integrity below S_crit_ [20].

### Gastric and Intestinal Blood Flows

Previous studies have either presented snapshots of gastrointestinal blood flow following brief exposures to hypoxia well within the postprandial period (e.g. 24 hr post feeding [7], [21], or focussed on the effects of moderate hypoxia during a critical swimming speed protocol, involving exposure and data collection for maximally a couple of hours [22]. Furthermore, only one study has so far measured long-term postprandial blood flows distal to the bifurcation of the coeliacomesenteric artery [21]. However, our study is the first to report continuous, high resolution (hourly basis) changes in both coeliac and mesenteric blood flow in response to feeding and hypoxic exposure. Following feeding there was an initial increase (by the third hour) in the blood flow through the gastric artery (coeliac) and a subsequent increase in the blood flow through the intestinal artery (mesenteric). This clear temporal pattern likely reflects the movement of the chyme within the gastrointestinal tract. In gadoid predators, the time *t*_end_ (h) for total evacuation (i.e. an empty stomach) of a meal depends upon the length of the predator (*L*, cm), the energy content (*E*, kJ g^−1^) and mass (*S*_0_, g) of the meal, and the temperature (*T*, °C). Using the parameterization of the square root model of gastric evacuation by Andersen [23], *t*_end_ (h) can be calculated as
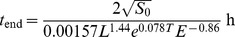



When calculating *t*_end_ (h) for each cod in normoxia a mean value of 32 h is obtained. In the present study the gastric blood flow was increased for 25 h in the normoxic group. This thus points to a tight correlation between the time it takes for the meal to be evacuated from the stomach and into the proximal intestine and the increased gastric blood flow.

### Possible Control Mechanisms

The present study confirms that both the gastric and the intestinal blood flow remains unaltered in fed fish when the ambient oxygen level is changed from normoxic to hypoxic (Fig. 2), with no long-term impact on the digestive efficiency. In contrast both blood flows decreased abruptly when unfed fish were exposed to hypoxia (Fig. 1). Besides hypoxic exposure, activity or struggling behavior may cause a shift in blood flow away from the gut and supposedly towards the skeletal muscle (reviewed by [24]). However, since we observed no, or very minor, behavioral responses when the fish were exposed to hypoxia, we conclude that both gastric and intestinal blood flows are responsive to a lowering of the oxygen level in unfed fish. This is in accordance with previous results obtained from measurements of the blood flow in the coeliacomesenteric artery of Atlantic cod and green sturgeon, *Acipenser medirostris* (Ayres) [7], [25], indicating that the gastrointestinal blood flow is of a low priority under hypoxic conditions in unfed animals. This disparity is best explained by the fact that local metabolic factors such as the tissue oxygen level, proton or ion content, dominate under postprandial conditions as has been shown in several studies in fish [7], [10], whereas a sympathetic tone on the resistance vessels, i.e. the arterioles, of the gastrointestinal tract regulates the gastrointestinal blood flow in unfed fish in accordance with the requirements of the somatic circulation [26], [27]. Consequently, under circumstances where certain tissues, especially those of substantial importance, have a high oxygen demand, local metabolic and vasoactive factors subdue the regulation via the central nervous system [27], [28], [29]. The fact that the postprandial gastrointestinal circulation is spared under moderate hypoxic conditions as a result of local control mechanisms, signifies the evolutionary importance of maintaining a sufficient oxygenation of this organ in order to efficiently digest and absorb the nutrients along the gastrointestinal canal.

### Correlations between the Measured Variables

In fish, feeding is almost always accompanied by a compensatory increase in CO [7], [21], [22], which in some species is accompanied by an additional redistribution of blood from the systemic circulation to the gut [26]. As expected CO increased following feeding and the tight coupling in the time course of CO and Q_me_ (except for the first few hours where CO and Q_ce_ were concomitantly elevated) suggest that CO in cod provides a significant contribution to the augmented oxygen requirements of the gastrointestinal tract. Initially, and corresponding to the time course of Q_ce_, this is supported by elevated HR. A similar clear positive relationship between postprandial HR and gastrointestinal blood flow has been shown in rainbow trout (*Oncorhynchus mykiss*), prevailing throughout the digestive period [30]. Such a correlation between Q_ce_ and HR would allow the use of HR biotelemetry to study feeding in free swimming fish (as also suggested by the authors [30]) and hence exclude the confounding effects of force-feeding on digestion [31]. However since the increased postprandial gastrointestinal blood flow in cod (returning to baseline levels 56 h after feeding) is only initially driven by increased HR (returning to baseline level 25 h after feeding) and subsequently shifts to rely on increased stroke volume, biotelemetry of CO or gastrointestinal blood flow seems the only viable solution in this species. This has until now only been done in the sturgeon [25], [32].

Postprandial metabolic rates remained elevated beyond the time that CO, Q_ce_, and Q_me_ returned to prefeeding rates. An explanation for the extended elevated MO_2_ is that the latter component of the SDA response is largely attributed to the postabsorptive processes of meal assimilation, including protein and glycogen synthesis (reviewed by [33]. In support, the infusion of amino acids into the bloodstream of the catfish, *Ictalurus punctatus*, increased metabolic rate, a response that was abolished by the administration of the protein synthesis inhibitor cycloheximide [34], [35]. Since CO returned to baseline well before postprandial MO_2_ declined completely (above), it is clear that the final metabolic demands of the SDA are met by augmented oxygen extraction, E_O2_.

### SDA

The pre-feeding (i.e. baseline) SMR values in the present study are highly comparable to previous reports by Schurmann and Steffensen [15] (35.5 mg O_2_ kg^−1^ h^−1^ 5°C) but higher than found by Steffensen et al. [36] at 4.5°C. With no differences between the SDA variables in normoxic and hypoxic fish (Table 1) it is clear that the fish in the present study had sufficient cardiorespiratory capacity to maintain the digestive processes even during and after the 90 minutes exposure to moderate hypoxia. Or, in other words, digestive efficiency was not significantly affected by moderate hypoxia. Overall however, the digestive efficiency (expressed as the cost of digestion, i.e. the SDA_coef_) of fish in our study was low compared to previous reports on cod. Jordan and Steffensen [18] found a SDA_coef_ that was 50% of that in the present study for fish fed a fish filet meal of comparable size. Their cod however fed voluntarily and had not undergone surgery while the lower efficiency in our study could be a consequence of the combined effects of surgical intervenes, instrumentation and force-feeding, factors which together influence the capacity to evacuate a meal [31]. The lower temperature in our study (6.5 versus 10°C in Jordan and Steffensen [18]) may be an additional explainable variable for the low efficiency. This is supported by a recent study on cod where a reduction in temperature from 11 to 6°C resulted in higher SDA coefficients [37].

We illustrate, using observed residence times in hypoxia [2], that Atlantic cod return to normoxia before the onset of any negative effects. Consequently growth and other vital parameters which are based on their energy uptake should not be compromised. It can however not be excluded that the increased MO_2_ following prey ingestion (i.e. the SDA) may compromise, or be compromised by, any further excursion(s) into hypoxic water while a meal is being digested. In other words, the fish may not be able to re-enter hypoxic water while digesting a previous meal, with negative impact on feeding frequency and hence growth. Nevertheless, this ability of a species to use sub-optimal environments to search for food, even at limited intensity, residence and search time, is probably vital to understand species interactions and their bioenergetic implications.

## References

[1] Conley DJ, Humborg C, Rahm L, Savchuk OP, Wulff F (2002) Hypoxia in the Baltic Sea and basin-scale changes in phosphorus biogeochemistry. Environmental Science & Technology 36: 5315–5320. 12521155 10.1021/es025763w

[2] Neuenfeldt S, Andersen KH, Hinrichsen H-H (2009) Some Atlantic cod Gadus morhua in the Baltic Sea visit hypoxic water briefly but often. J Fish Biol 75: 290–294. 20738498 10.1111/j.1095-8649.2009.02281.x

[3] Schurmann H, Steffensen JF (1992) Lethal oxygen levels at different temperatures and the preferred temperature during hypoxia of the Atlantic cod, *Gadus morhua* L. J Fish Biol. 41: 927–934.

[4] Plante S, Chabot D, Dutil JD (1998) Hypoxia tolerance in Atlantic cod. J Fish Biol 53: 1342–1356.

[5] Neuenfeldt S, Beyer JE (2003) Oxygen and salinity characteristics of predator–prey distributional overlaps shown by predatory Baltic cod during spawning. J Fish Biol 62: 168–183.

[6] Prince ED, Phillip C (2006) Hypoxia-based habitat compression of tropical pelagic fishes. Fisheries Oceanography 15: 451–464.

[7] Axelsson M, Fritsche R (1991) Effects of exercise and feeding on the gastrointestinal blood flow in the Atlantic cod *Gadus morhua* . J Fish Biol 158: 181–198. 10.1242/jeb.158.1.181 1717628

[8] Fritsche R, Axelsson M, Franklin CE, Grigg GG, Holmgren S, et al (1993) Respiratory and cardiovascular responses to hypoxia in the Australian lungfish. Respiratory Physiology 94: 173–187. 10.1016/0034-5687(93)90046-d 8272589

[9] Thorarensen H (1994) Ph.D. thesis. Simon Fraser University,Burnaby, BC, Canada.

[10] Axelsson M, Altimiras J, Claireaux G (2002) Post-prandial blood flow to the gastrointestinal tract is not compromised during hypoxia in the sea bass *Dicentrarchus labrax* . J Exp Biol 205: 2891–6. 12177153 10.1242/jeb.205.18.2891

[11] Priede IG (1977) Natural selection for energetic efficiency and the relationship between activity level and mortality. Nature (London) 267: 610–610. 876379 10.1038/267610a0

[13] Lewis JM, Costa I, Val AL, Almeida-Val VMF, Gamperl AK, et al (2007) Responses to hypoxia and recovery: repayment of oxygen debt is not associated with compensatory protein synthesis in the Amazonian cichlid, *Astronotus ocellatu* . J Exp Biol 210: 1935–1943. 17515419 10.1242/jeb.005371

[14] Svendsen JC, Steffensen JF, Aarestrup K, Frisk M, Etzerodt A, et al (2011) Excess posthypoxic oxygen consumption in rainbow trout (*Oncorhynchus mykiss*): recovery in normoxia and hypoxia. Canadian J Zool 90: 1–11.

[15] Thuy NH, Tien LA, Tuyet PN, Huong DTT, Cong NV, et al.. (2010) Critical oxygen tension increases during digestion in the perch *Perca fluviatilis*. J Fish Biol 76, 1025–1031.

[16] Schurmann H, Steffensen JF (1997) Effects of temperature, hypoxia and activity on the metabolism of juvenile Atlantic cod. J Fish Biol 50: 166–1180.

[17] Secor SM (2009) Specific dynamic action: a review of the postprandial metabolic response. J Comp Physiol B 179: 1–56. 18597096 10.1007/s00360-008-0283-7

[18] Gnaiger E (1983) Calculations of energetic and biochemical equivalents of respirometry oxygen consumption. In: Gnaiger E, Forstener H, editors. Polarographic Oxygen Sensors. Springer, Berlin. 337–345.

[19] Jordan AD, Steffensen JF (2007) Effects of ration size and hypoxia on specofoc dynamic action in cod. Physiol Biochem Zool 80: 178–185. 17252514 10.1086/510565

[20] Gamperl AK, Driedzic WR (2009) Cardiovascular Responses to Hypoxia. In: Fish Physiology, Vol. XXVII. Richards J, editor. Brauner CJ, Farrell AP series editors. Academic Press. 302–360.

[21] McKenzie DJ, Skov PV, Taylor EWT, Wang T, Steffensen JF (2009) Abolition of reflex bradycardia vagotomy has no effect on the regulation of oxygen uptake by Atlantic cod in progressive hypoxia. Comp Biochem Phys A 153: 332–338. 10.1016/j.cbpa.2009.03.009 19303050

[22] Axelsson M, Thorarensen H, Nilsson S, Farrell AP (2000) Gastrointestinal blood flow in the red Irish lord, *Hemilepidotus hemilepidotus*: long-term effects of feeding and adrenergic control. J. Comp. Physiol. B. 170: 145–152. 10.1007/s003600050269 10791574

[23] Dupont-Prinet A, Claireaux G, McKenzie D J (2009) Effects of feeding and hypoxia on cardiac performance and gastrointestinal blood flow during critical speed swimming in the sea bass *Dicentrarchus labrax* . Comp Biochem Phys A 154: 33–40. 10.1016/j.cbpa.2009.06.015 19559805

[24] Andersen NG (2001) A gastric evacuation model for three predaotory gadoids and implications of using pooled field data of stomach contents to estimate food rations. J Fish Biol 59: 1198–1217.

[25] Farrell AP, Thorarensen H, Axelsson M, Crocker CE, Gamperl AK, et al (2001) Gut blood flow in fish during exercise and severe hypercapnia. Com Biochem Physiol A 128: 549–561. 10.1016/s1095-6433(00)00335-4 11246044

[26] Gräns A, Axelsson M, Pitsillides K, Olsson C, Höjesjö J, et al (2009) A fully implantable multi-channel biotelemetry system for measurement of blood flow and temperature: a first evaluation in the green sturgeon. Hydrobiologia 619: 11–25.

[27] Seth H, Sandblom E, Holmgren S, Axelsson M (2008) Effects of gastric distension on the cardiovascular system in rainbow trout (*Oncorhynchus mykiss*) Am J Physiol Regul Integr Comp Physiol. 294: R1648–R1656. 10.1152/ajpregu.00900.2007 18337308

[28] Seth H, Axelsson M (2010) Sympathetic and parasympathetic regulation of the postprandial gastrointestinal hyperemia in rainbow trout (*Oncorhynchus mykiss*). J Exp Biol 213: 3118–3126. 20802112 10.1242/jeb.043612

[29] Seth H, Gräns A, Axelsson M (2010) Cholecystokinin (CCK) as a potentially important regulator of the postprandial gut blood flow in rainbow trout (*Oncorhynchus mykiss*). Am J Physiol Regul Integr Comp Physiol 298: 1240–1248. 10.1152/ajpregu.00781.2009 20164206

[30] Seth H, Axelsson M, Farrell AP (2011) The circulation and metabolism of the gastrointestinal tract (Chapter 9). In: Fish Physiology Volume 30. The Multifunctional Gut of Fish. Grosell M, Farrell AP, Brauner CJ editors. Elsevier Science & Technology, Academic Press. Pp. 351–393.

[31] Eliason EJ, Higgs DA, Farrell AP (2008) Postprandial gastrointestinal blood flow, oxygen consumption and heart rate in rainbow trout (*Oncorhynchus mykiss*). Comp Biochem Physiol A: 149, 380–388. 10.1016/j.cbpa.2008.01.033 18308602

[32] Behrens JW, Gräns A, Andersen NG, Neuenfeldt S, Axelsson M (2011) Recovery of gastric evacuation rate in Atlantic cod *Gadus morhua* L surgically implanted with a dummy telemetry device. Laboratory Animals 45: 240–246. 21771807 10.1258/la.2011.011013

[33] Gräns A, Olsson C, Pitsillides K, Nelson HE, Cech JJ Jr, et al (2010) Effects of feeding on thermoregulatory behaviours and gut blood flow in white sturgeon (*Acipenser transmontanus*) using biotelemetry in combination with standard techniques. J Exp Biol 213: 3198–206. 20802122 10.1242/jeb.043570

[34] Carter CG, Houlihan DF (2001) Protein synthesis. In Wright PA, Andersen PM, editors. Nitrogen Excretion. Academic Press, San Diego, CA. 31–75.

[35] Brown CR, Cameron JN (1991a) The induction of specific dynamic action in channel catfish by infusion of essential amino acids. Physiol Zool 64: 276–297.

[36] Brown CR, Cameron JN (1991b) The relationship between specific dynamic action (SDA) and protein synthesis rates in the channel catfish. Physiol Zool 64: 298–309.

[37] Steffensen JF, Bushnell PG, Schurmann H (1994) Metabolic rate of 4 Arctic species of teleosts from Greenland. Polar Biol 14: 49–54.

[38] Pérez-Casanova JC, Santosh PL, Gamperl AK (2010) Effects of dietary protein and lipid level, and water temperature, on the post-feeding oxygen consumption of Atlantic cod and haddock. Aquaculture Research 41: 198–209.

